# Bone-Specific Overexpression of PITX1 Induces Senile Osteoporosis in Mice Through Deficient Self-Renewal of Mesenchymal Progenitors and Wnt Pathway Inhibition

**DOI:** 10.1038/s41598-019-40274-6

**Published:** 2019-03-05

**Authors:** Nancy Karam, Jean-François Lavoie, Benoit St-Jacques, Saadallah Bouhanik, Anita Franco, Nihad Ladoul, Alain Moreau

**Affiliations:** 1 Viscogliosi Laboratory in Molecular Genetics of Musculoskeletal Diseases, Sainte-Justine University Hospital Research Center, Montréal, Québec H3T 1C5 Canada; 2 Department of Biochemistry and Molecular Medicine, Faculty of Medicine, Université de Montréal, Montréal, Québec H3T 1J4 Canada; 3 Department of Stomatology, Faculty of Dentistry, Université de Montréal, Montréal, Québec H3T 1J4 Canada

## Abstract

The cellular and molecular mechanisms underlying senile osteoporosis remain poorly understood. In this study, transgenic *mCol1α1-Pitx1* mice overexpressing paired-like homeodomain 1 (PITX1), a homeobox transcription factor, rapidly develop a severe type-II osteoporotic phenotype with significant reduction in bone mass and biomechanical strength similar to that seen in humans and reminiscent of the phenotype previously observed in *Sca-1* (*Ly6a*)-null mice. PITX1 plays a critical role in hind limb formation during fetal development, while loss of expression is associated with primary knee/hip osteoarthritis in aging humans. Through *in vivo* and *in vitro* analyses, we demonstrate that *Pitx1* directly regulates the self-renewal of mesenchymal progenitors and indirectly regulates osteoclast differentiation through the upregulation of Wnt signaling inhibitors *DKK1*, *SOST*, and *GSK3-β*. This is confirmed by elevated levels of plasma DKK1 and the accumulation of phospho-β-catenin in transgenic mice osteoblasts. Furthermore, overexpressed *Pitx1* in mice osteoblasts results in severe repression of *Sca-1* (*Ly6a*) that was previously associated with senile osteoporosis. Our study is the first to demonstrate the novel roles of PITX1 in senile osteoporosis where PITX1 regulates the self-renewal of mesenchymal stem cells or progenitor cells through *Sca-1 (Ly6a)* repression and, in addition, inhibits the Wnt signaling pathway.

## Introduction

The mechanisms underlying bone formation and bone loss are not fully understood in different pathological conditions. Several studies suggest a role for the transcription factor PITX1 in bone metabolism and in age-related/dependent osteoporosis (type-II osteoporosis). Previous comparative gene expression analysis of osteoblasts derived from 72-year-old monozygotic twins discordant for osteoporosis reported an 8.6-fold upregulation of *Pitx1* expression in primary osteoblast cells from the osteoporotic twin when compared to the non-osteoporotic twin^1^.

PITX1 is one of three proteins in the homeobox transcription factor family (PITX1, PITX2, and PITX3)^2^. During mouse fetal development, *Pitx1* is highly expressed in the perichondrium of hind limb long bones, suggesting its importance in skeletal and articular joint development^3–5^. Interestingly, aging *Pitx1*+/− heterozygous mice that are normal at birth progressively develop osteoarthritis-like lesions in their hip/knee cartilage along with a drastic increase in cortical and trabecular bone formation^6^. Loss of *Pitx1* expression was also reported in articular cartilage of humans suffering from knee or hip osteoarthritis^6^ through a transcriptional mechanism involving nuclear accumulation of prohibitin^7^. Since the partial loss-of-function of *Pitx1* causes an increase in bone formation and density, it is conceivable that its gain-of-function could have the opposite effect, possibly causing an osteoporotic-like phenotype.

In the present study, we show that transgenic *mCol1α1-Pitx1* mice exhibit a phenotype reminiscent to human-related (type-II) osteoporosis with reduced bone mineral density (BMD) and increased susceptibility to fractures. Unlike postmenopausal (type-I) osteoporosis that results from an imbalance towards bone resorption, *mCol1α1-Pitx1* mice have both decreased bone formation from an osteoprogenitor deficiency and decreased bone resorption as a consequence of the inhibition of the Wnt/β-catenin signaling pathway. The decrease in osteoprogenitors in *mCol1α1-Pitx1* adult mice is not directly due to defects in osteoblastogenesis but rather due to reduced self-renewal activity of multilineage mesenchymal progenitors. Our data suggest that *Pitx1* overexpression reduces the self-renewal of the mesenchymal stem cell population through the repression of *Sca-1/Ly6a*.

## Results

### Generation of transgenic *mCol1α1-Pitx1* mice

**Figure 1:**
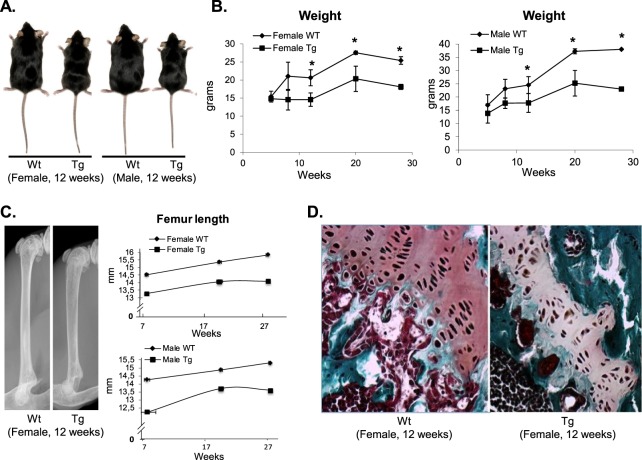
Transgenic *Col1α1-Pitx1* mice exhibit growth retardation accompanied with bone loss. (**A**) Comparative photo of both sexes from 12-week-old transgenic *mCol1α1-Pitx1* and wild type mice. (**B**) Comparative growth curves of both sexes of *mCol1α1-Pitx1* mice and their corresponding wild type littermates over a 28-week period. (**C**) X-ray imaging of 12-week-old female *mCol1α1-Pitx1* and wild type mice confirm a very thin and fragile cortical bone in long bones. The femur length of both sexes of transgenic *mCol1α1-Pitx1* mice and their corresponding wild type littermates over a 28-week period are also shown. (**D**) Histological examination of the distal femoral growth plates for the study of growth differences of long bones. Structural abnormalities of growth plates were observed in safranin O-stained sections of transgenic *mCol1α1-Pitx1* mice. Mean proteoglycan content in transgenic growth plates was determined by measuring changes in safranin O color intensity using the Image J software. Proteoglycan content of transgenic mouse growth plates showed a reduction of 60% when compared to the wild type mouse growth plate.

A transgene composed of the 2.3 kb fragment of the *mColIα1* promoter, which controls the expression of the *Pitx1* coding sequence (murine *Pitx1* cDNA), was constructed in a pCI plasmid. A synthetic intron separates the promoter from the coding sequence and a polyadenylation cassette follows the coding sequence in this construct. The transgenic mice were generated at the IRIC Transgenesis Platform (Université de Montréal) and were subsequently maintained at CHU Sainte-Justine Research Center. The transgenic mice were tested to determine the expression level of the transgene. Quantitative real time PCR (qPCR) was used to measure the level of expression using RNA extracted from tail biopsies. Transgenic lines were established from mice M22, F30, M42, and M51. Mice of line 30 exhibited the highest *Pitx1* expression levels (~5-fold compared to wild type mice), while transgenic lines 22, 42, and 51, displayed lower levels of *Pitx1* expression (~1.8 fold), compared to wild type mice. Therefore, all our analyses were conducted with the transgenic line F30. The transgenic *mCol1α1-Pitx1* mice were smaller than their wild type littermates (Fig. 1A). The body weights of the transgenic mice were significantly reduced by 28.7% and 39.5% in females (*P* = 0.006) and males (*P* = 0.001), respectively compared with wild type mice (Fig. 1B).

### *Pitx1* overexpression reduces bone length and bone mass

Radiological analysis revealed a 10.7% and 11.1% reduction in femur length of adult transgenic mice for females (P = 0.01) and males (P = 0.007), respectively (Fig. 1C). Given the central role of chondrocyte proliferation and differentiation in the growth of long bones, histological examination of the distal femoral growth plates was performed. Structural abnormalities of growth plates were observed in safranin O-stained sections of transgenic *mCol1α1-Pitx1* mice (Fig. 1D). The proliferation and hypertrophy zones were narrower than in the wild type mice. The proliferation zone displayed visibly lower number of cells, and this was accompanied with irregular chondrocyte organization that failed to form distinct columns. In addition, chondrocytes appeared smaller and isolated in a poorly stained matrix. ImageJ software was used to quantify the intensity of safranin O staining and showed 59% reduction in the proteoglycan content when compared to the wild type littermates (wild type [mean ± SD, arbitrary units]: 95.7 ± 8.8 and transgenic: 39.3 ± 9.5).

Bone mineral density (BMD) measurements of whole femurs of transgenic and wild type mice showed a 15.9% and 13.4% reduction in transgenic females (*P* = 0.014) and transgenic males (*P* = 0.023) respectively, compared with wild type mice at 12 weeks of age (Supplementary Fig. S1). Similarly, the bone mineral content (BMC) was reduced by 13.3% in females (*P* = 0.05), and 23.3% in males (*P* = 0.05) compared with wild type mice (Supplementary Fig. S1). The decrease in bone mass was observed in females throughout the 28-week observation period. In transgenic males, the bone mass was decreased prior the first 20 weeks of observation and then became similar to that of wild type mice. These observations indicate that bone mass is reduced with *Pitx1* overexpression in mice osteoblasts.

### *Pitx1* overexpression impairs bone microarchitecture and biomechanical strength

**Figure 2:**
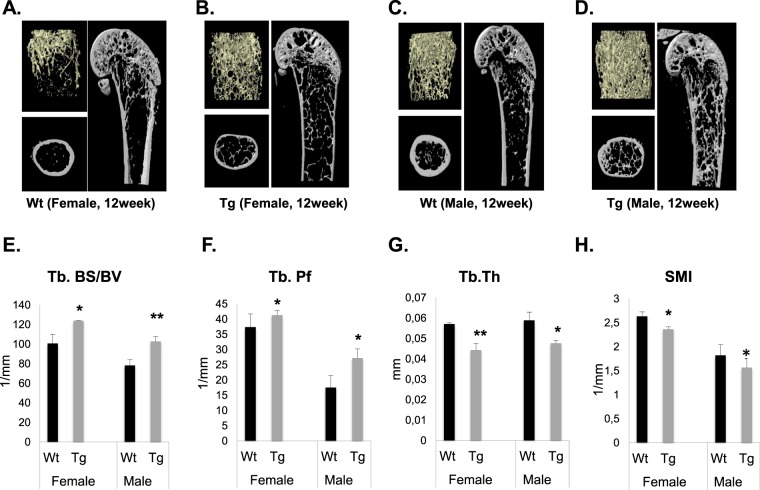
Transgenic *Col1α1-Pitx1* mice exhibit an altered trabecular microarchitecture. (**A**–**D**) Cross-sectional microCT scans of femurs from both sexes of 12-week-old *Col1α1-Pitx1* and wild type (WT) mice. (**E–H**) Quantitative comparison of the trabecular bone parameters: Bone surface (BS)/Bone volume (BV); pattern factor (Pf); thickness (Th); structure model index (SMI). Data are presented as mean ± SD for 5 mice from each sex in each genotype group. Asterisks indicate statistically significant difference (*p < 0.05; **p < 0.005; ***p < 0.0005).

**Figure 3:**
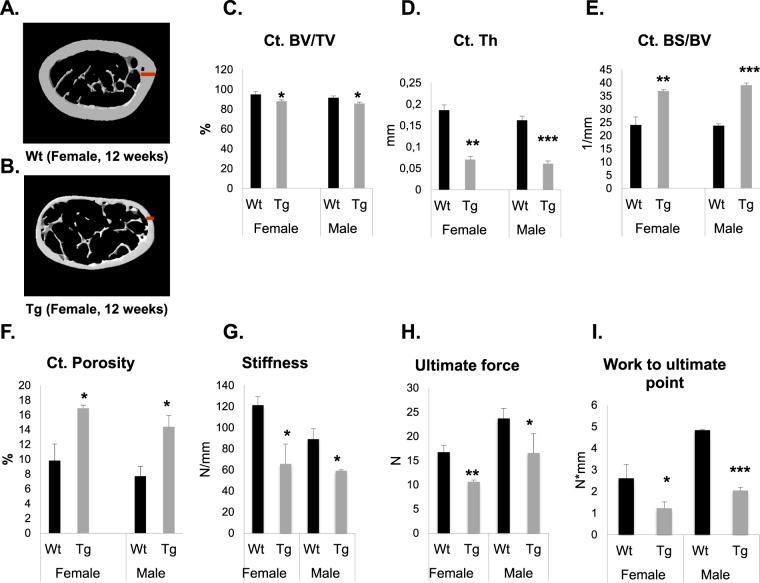
Transgenic *Col1α1-Pitx1* mice exhibit altered cortical microarchitecture accompanied with a loss of bone strength. (**A–D**) Representative cross-sectional microCT scans of femurs from females of 12-week-old transgenic *mCol1a1-Pitx1* and wild type mice. (**C–F**) Quantitation of the cortical bone – Bone volume (BV)/trabecular volume (TV); Bone surface (BS)/BV; Porosity (Th). (**G–I**) Three-point bending test quantitation – Stiffness; Ultimate Force; Work to ultimate point. Data are presented as mean ± SD for 5 mice from each sex in each genotype group. Asterisks indicate statistically significant difference (*p < 0.05; **p < 0.005; ***p < 0.0005).

To determine the physiological effects of decreased mineral content in transgenic *mCol1α1-Pitx1* mice, structural and mechanical analyses were performed. Microcomputed tomography (μCT) was used to analyze the structural parameters of the trabecular and cortical bones of femurs harvested from 12-week-old transgenic and wild type mice of both sexes. Three-dimensional images showed alterations in the trabecular bones of transgenic mice when compared to those of wild type mice (Fig. 2A–D). Quantitation of the structural parameters revealed that the trabecular bone surface over bone volume (Tb.BS/BV) and the trabecular pattern factor (Tb.Pf) were increased by 23.1% and 10.4%, respectively, in transgenic females (*P* < 0.01), and increased by 31.4% and 55.4%, respectively, in transgenic males (*P* < 0.01) (Fig. 2E,F). Trabecular pattern factor (Tb.Pf) describes the relative convexity/concavity of the total bone surface and is the reciprocal measurement of trabecular network connectivity^8^. Indeed, increased Tb.Pf values in transgenic mice represent the presence of isolated, disconnected structures (struts) and reflect a weak trabecular network (Fig. 2F). Conversely, trabecular thickness (Tb.Th) was decreased by 22.8% in transgenic females (*P* < 0.01) and by 19% (*P* < 0.01) in transgenic males (Fig. 2G). Additionally, the structure model index (SMI), used to determine plate or rod-like geometry of trabecular structures^9^, also decreased by 11.5% in transgenic females (*P* < 0.01) and by 16.7% (*P* < 0.01) in transgenic males (Fig. 2H), indicating a shift from rod-like trabecular architecture to a plate-like one. Trabecular bone volume (Tb.BV/TV) and trabecular number (Tb.N) were also measured in 12 week-old wild type and transgenic mice, but they showed no significant difference in males (*P* = 0.09 and *P* = 0.340, respectively) or females (*P* = 0.293 and *P* = 0.080, respectively) (Supplementary Fig. S2). Quantitation of the structural parameters in cortical bones revealed that the cortical bone volume density (Ct.BV/TV) and the cortical thickness (Ct.Th) were decreased by 7.2% and 55.6%, respectively, in transgenic females (*P* < 0.01) and by 6.5% and 62.5%, respectively, in transgenic males (*P* < 0.01) (Fig. 3A–D) compared to the wild type mice. Conversely, the cortical bone surface over bone volume (Ct.BS/BV) and the cortical porosity (Ct.porosity) were significantly increased in transgenic mice by 54% and 81.6%, respectively, in females (*P* < 0.01) and by 64.4% and 87%, respectively, in males (*P* < 0.01) (Fig. 3E–F). The biomechanical strength of freshly dissected femurs was evaluated by a three-point bending test. Consistent with the BMC and BMD data, the three-point bending test of femurs revealed a decrease in the bone strength in transgenic mice by 45.8% in females (*P* = 0.03) and 33.8% in males (*P* = 0.025) compared to wild type mice (Fig. 3G). The ultimate force parameter was significantly reduced in femurs isolated from transgenic mice (10.6 ± 0.3 N, *P* < 0.005 in females and 16.5 ± 4 N, *P* < 0.05 in males) compared to wild type mice (16.7 ± 1.4 N in females and 23.7 ± 2 N in males) (Fig. 3H). The work to ultimate point was significantly reduced by 53.8% in transgenic females (*P* = 0.05) and by 58.3% in transgenic males (*P* = 0.0007) compared to wild type mice (Fig. 3I). Collectively, our data reveal that adult transgenic mice overexpressing *Pitx1* in bone tissues have reduced bone mass density, compromised microarchitecture, and reduced biomechanical bone strength compared with wild type mice.

### Changes in bone formation in transgenic *mCol1α1-Pitx1* mice

**Figure 4:**
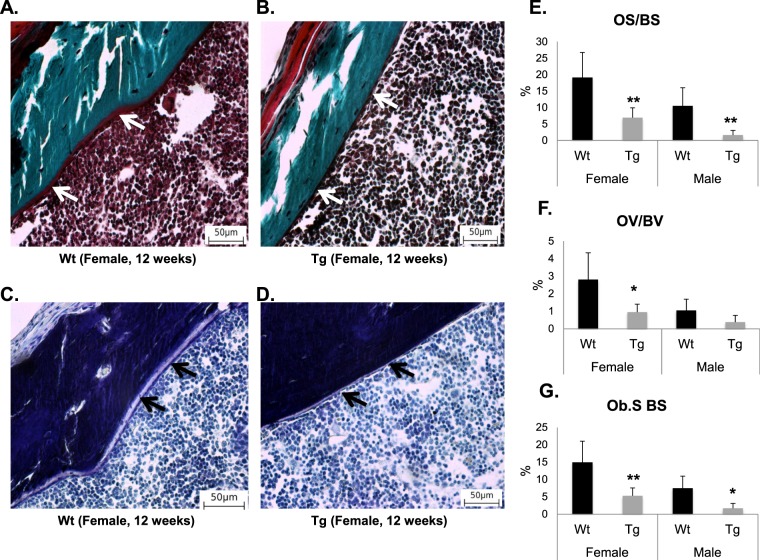
Impaired *in vivo* osteoblast number derived from 12-week-old transgenic *mCol1α1-Pitx1* mice. (**A–D**) Representative Goldner and toluidine blue staining on femur sections reveal a decreased number of osteoblasts and osteoids in transgenic female *mCol1α1-Pitx1* mice compared to wild type female mice. (**E–G**) Histomorphometric quantitation of the osteoid surface (OS/BS), volume (OV/BV), and osteoblast surface (Ob.S BS). Data are presented as mean ± SD for 5 mice from each sex in each genotype group. Asterisks indicate statistically significant difference (*p < 0.05; **p < 0.005).

To determine whether the reduced bone mass in transgenic mice was due to decreased bone formation, we performed static and dynamic bone histomorphometry analysis on sections of undecalcified femurs of wild type and transgenic mice at 12 weeks of age. Goldner’s trichrome and toluidine blue staining revealed reduced number of osteoids in the cortical bones of transgenic mice (Fig. 4A–D). Transgenic mice exhibited a significant decrease in trabecular perimeters occupied by osteoids, presented as the ratio of the osteoid surface to bone surface (OS/BS), with a reduction of 64.2% in females (*P* = 0.003) and 85% in males (*P* = 0.007) (Fig. 4E). In addition, transgenic mice displayed a significant decrease in the osteoid volume over bone volume (OV/BV) only in females (67.9%, *P* = 0.012), while such decrease nearly approached significance in males (67.3%, *P* = 0.054) (Fig. 4F). Among bone formation parameters, the number of osteoblasts lining the trabecular bone (N.Ob/B.Pm) and the percentage of bone surface occupied by osteoblasts (Ob.S/BS) significantly decreased by 64.4% in transgenic females (*P* = 0.003) and 77.3% in transgenic males (*P* = 0.007) compared with wild type mice (Fig. 4G). We also observed a significant reduction in the mineralized surface, bone formation rate, and mineral appositional rate in transgenic mice as demonstrated by dynamic histomorphometric analysis of femurs from 12-week-old transgenic *mCol1α1-Pitx1* and wild type mice (Supplementary Fig. S3). Indeed, the bone formation rate (BFR/BS), mineral apposition rate (MAR), and the mineralizing surface (MS/BS) were all significantly reduced in transgenic mice compared with wild type mice (Supplementary Fig. S3). Our data demonstrate that *Pitx1* overexpression induces altered mineralization, defects in bone formation and osteoid deposition, resulting in low BMD and altered microarchitecture in transgenic mice.

### Overexpression of *Pitx1* affects osteoblast differentiation and mineralization

**Figure 5:**
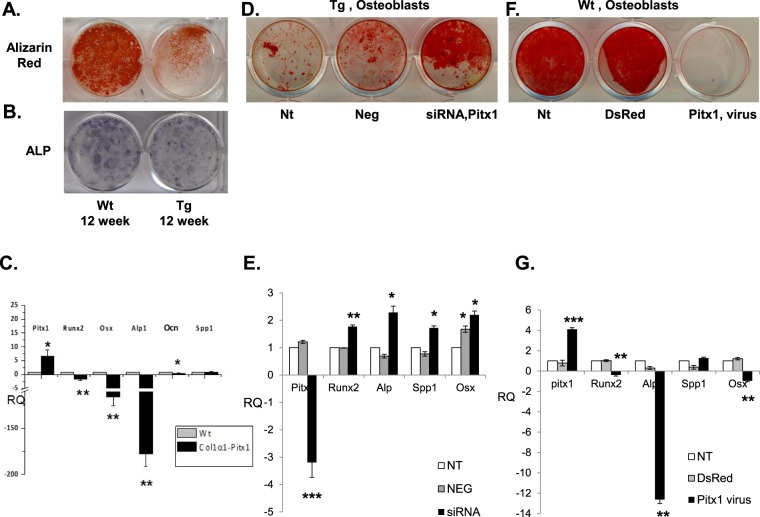
Impaired *in vitro* osteoblast differentiation derived from 12-week-old transgenic *mCol1α1-Pitx1* mice when compared to their wild type littermates. (**A**,**B**) Alizarin red and ALP staining of osteoblast cultures after 14 days of induction in osteogenic medium. (**C**). Real time PCR analysis of *Pitx1* and osteogenic markers (*Runx2*, *Osx*, *Alp1*, *Ocn* and *Spp1*) in wild type (Wt) and transgenic (*mCol1α1-Pitx1*) osteoblasts on day 14 of osteogenic induction. Expression was normalized with that of wild type. Asterisks indicate statistically significant difference (*P < 0.05; **P < 0.005). (**D**) Alizarin red staining of osteoblast culture after transfection with either siRNA targeting Pitx1 (siRNA Pitx1) or scrambled sequence siRNA (Neg) used as a control. (**E**) Real time PCR analysis of *Pitx1* and osteogenic markers (*Runx2*, *Osx*, *Alp1 and Spp1*) in mouse transgenic osteoblasts untransfected (NT), transfected with *Pitx1* siRNA or with a scramble sequence siRNA (Neg). Expression was normalized with that of untransfected transgenic osteoblasts. Asterisks indicate statistically significant difference (*P < 0.05; **P < 0.005). (**F**) Alizarin red staining of osteoblast culture after infection with either lentivirus coding for Pitx1 or DsRed as a control. (**G**) Real time PCR analysis of *Pitx1* and osteogenic markers (*Runx2*, *Osx*, *Alp1* and *Spp1*) in wild type mouse osteoblasts uninfected (NT), infected with either lentivirus coding for *Pitx1* or DsRed as a control. Expression was normalized with that of uninfected wild type osteoblasts. Asterisks indicate statistically significant difference (*P < 0.05; **P < 0.005; ***P < 0.0005).

To investigate changes in osteoblast differentiation, we used alkaline phosphatase, which is correlated to bone formation, and alizarin red, a fluorescent dye for the detection of mineralized nodules in osteoblasts. We compared the formation of alkaline phosphatase (ALP)-positive colonies and the number of mineralized nodules (alizarin red staining) in primary osteoblast cultures derived from wild type and transgenic mice. Overexpression of *Pitx1* in transgenic mice osteoblasts caused a reduction in the number of ALP-positive cells and mineralized nodules compared with wild type mouse osteoblasts over a 21-day culture period (Fig. 5A,B). Surfaces of mineralized nodules stained with alizarin red, estimated by ImageJ software, were at 95.7% and 39.7% in wild type and transgenic mouse osteoblasts respectively (wild type [mean ± SD, arbitrary units]: 14861 ± 6.8 and transgenic: 6170 ± 10.9) (Fig. 5A). We then examined the expression of genes related to osteoblast differentiation. The expression level of differentiation markers *Runx2*, *Osx*, *Alp*, and *Ocn* were significantly reduced in transgenic cells compared with wild type mice (Fig. 5C). Conversely, silencing *Pitx1* expression in transgenic osteoblasts transfected with *Pitx1*-siRNA, was sufficient to rescue transgenic osteoblast differentiation and mineralization defects as well as to induce upregulation of tested osteoblast differentiation marker expression (Fig. 5D,E). Indeed, mineralized nodules were increased in transgenic osteoblasts transfected with siRNA targeting Pitx1 with an alizarin red stained surface at 82.5% (siRNA Pitx1 [mean ± SD, arbitrary units]: 12860 ± 7.5) compared to 29.5% in transgenic osteoblasts transfected with a scrambled sequence siRNA (Neg [mean ± SD, arbitrary units]: 4580 ± 3.4) and 22% in untransfected transgenic mice osteoblasts (Nt [mean ± SD, arbitrary units]: 3416 ± 9.2) (Fig. 5D). Of note, *Pitx1* overexpression in wild type mouse osteoblasts infected with a lentivirus expressing *Pitx1* led to a 4.1-fold increase in *Pitx1* expression when compared to normal endogenous *Pitx1* expression or cells infected with the empty lentivirus, and reproduced the effects observed in transgenic osteoblasts (Fig. 5F,G). Consistently, the surfaces of mineralized nodules stained with alizarin red were significantly decreased in lentivirus-infected wild type osteoblasts overexpressing *Pitx1* with a surface estimated by ImageJ software at 0.9% (Pitx1 virus [mean ± SD, arbitrary units]: 3 ± 1.4) contrasting with the surface values of 92.8% obtained with either the wild type osteoblasts infected with the empty vector (DsRed [mean ± SD, arbitrary units]: 14405 ± 9.4) or 98.8% obtained with the uninfected osteoblasts (Nt [mean ± SD, arbitrary units]: 15842 ± 8.5) (Fig. 5F). Collectively, our results show that *Pitx1* overexpression induces cell-autonomous differentiation and mineralization defects in primary mouse osteoblasts.

### Transgenic *mCol1α1-Pitx1* mice exhibit a defect in mesenchymal progenitors

**Figure 6:**
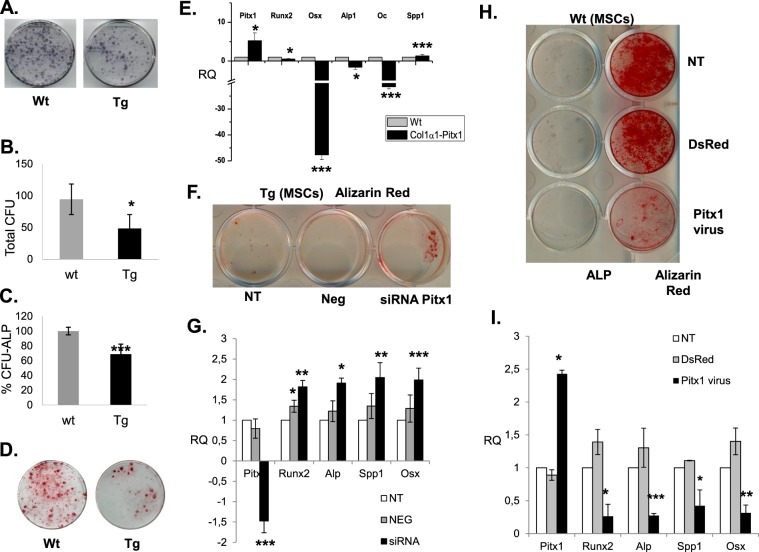
Mesenchymal stem cells (MSC) derived from 12-week-old transgenic *mCol1α1-Pitx1* mice display impaired osteoblast differentiation *in vitro* when compared to their wild type littermates. (**A**,**B**,**D**) Analysis of the colony-forming unit (CFU) number and osteogenic CFU-F number (ALP + CFU-F). (**C**) Alizarin red staining of MSC cultures after 14 days of induction in osteogenic media. (**E**) Real time PCR analysis of osteogenic markers (*Runx2*, *Osx*, *Alp1*, *Ocn* and *Spp1*) in MSCs. (**F**) Alizarin red staining of MSC culture after transfection with either siRNA targeting *Pitx1* or scrambled sequence siRNA (used as a control). H. Alizarin red staining of MSC culture after infection with either lentivirus coding for *Pitx1* or DsRed (control). (**G**,**I**) Real time PCR analysis of osteogenic markers (*Runx2*, *Osx*, *Alp1*, and *Spp1*) in MSCs. Expression was normalized with that of wild type.

To determine the origin of osteoprogenitor deficiency, equal numbers of nucleated bone marrow (BM) cells from 12-week-old transgenic and wild type mice were cultured. The number of colony forming units (CFU-F), the measure of total mesenchymal precursors including stem cells and committed progenitors formed in transgenic *mCol1α1-Pitx1* mice cultures compared to wild type cultures was reduced by 48.8% (*P* = 0.012) (Fig. 6A,B). These data demonstrate that mesenchymal precursors are fewer in transgenic mice compared to wild type mice. Furthermore, CFU-ALP was also decreased in transgenic mesenchymal stem cells (MSCs) in osteogenic differentiation media, exhibiting a 31.4% reduction (*P* = 0.0006) in the number of osteogenic ALP + (CFU-ALP+) colonies, few mineralized nodules, and a downregulation of osteogenic markers compared with wild type MSCs under the same conditions (Fig. 6C–E). Conversely, silencing of *Pitx1* expression by siRNA in transgenic MSCs decreased *Pitx1* expression by 3.2-fold when compared to untransfected transgenic cells (NT) or those transfected with scrambled siRNA (Neg). Reduced *Pitx1* expression levels in transgenic MSCs transfected with siRNA (siRNA *Pitx1*) increased their capacity to differentiate to mature osteoblasts as shown by the increase in mineralized nodules stained by alizarin red (4.5%) contrasting with values obtained with untransfected transgenic cells (NT - 0.6%) or those transfected with scrambled siRNA (Neg - 0.2%) (Fig. 6F). Similarly, the mRNA expression levels of osteogenic markers *Runx2*, *Osx*, *Alp and Spp1*, were elevated after silencing *Pitx1* expression in transgenic osteoblasts (Fig. 6G). Of note, *Pitx1* overexpression in lentivirus-infected wild type MSCs, increased *Pitx1* expression by 2.4-fold compared to uninfected cells and reproduced the effects observed in transgenic MSCs (Fig. 6H,I). Indeed, surfaces of mineralized nodules stained with alizarin red were decreased in lentivirus-infected wild type osteoblasts overexpressing *Pitx1* with a stained surface of 6%, in contrast with the mineralized surface values obtained with either the wild type osteoblasts infected with the empty vector (DsRed - 73.4%) or the uninfected osteoblasts (Nt – 81.1%) (Fig. 6H).

**Figure 7:**
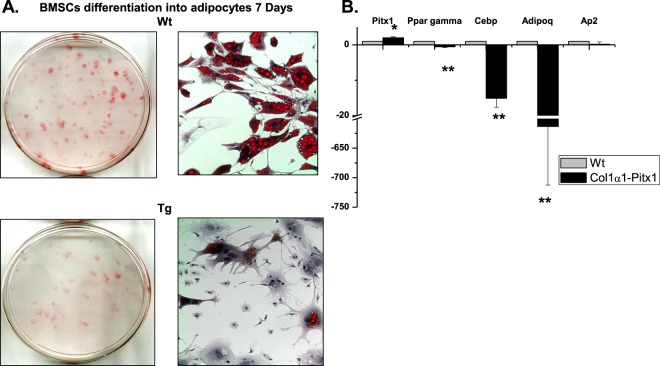
MSCs derived from 12-week-old transgenic *mCol1α1-Pitx1* mice displayed impaired adipocyte differentiation *in vitro* when compared to their wild type littermates. (**A**) Oil red ‘O’ staining of MSC cultures after 11 days of induction in adipogenesis media. (**B**) Real time PCR analysis of adipocytic markers (*Pparγ*, *Cebp*, *Adipoq* and *aP2*) in MSCs. Expression in real time PCR analysis was normalized in comparison with wild type. Asterisks indicate statistically significant difference (*p < 0.05; **p < 0.005).

**Figure 8:**
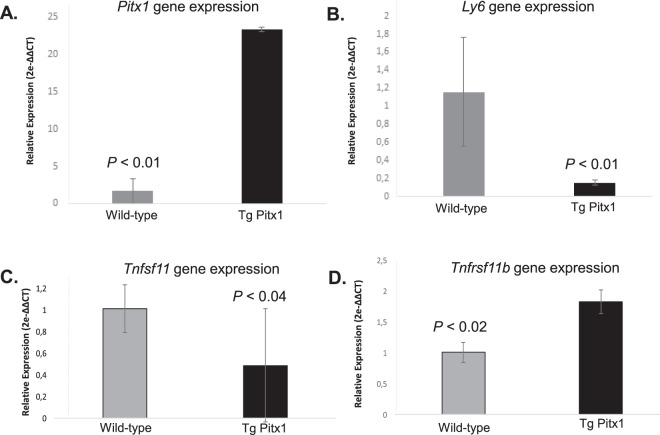
Expression analysis of *Pitx1*, *Sca-1(Ly6a)*, *Tnfsf11* and *Tnfrsf11b* gene in transgenic *mCol1α1-Pitx1* and wild type mouse primary osteoblasts. Real time qPCR analysis of *Pitx1* (**A**), *Sca-1 (Ly6a)* (**B**), *Tnsfs11* (**C**) and *Tnfrsf11b* (**D**) in transgenic *mCol1α1-Pitx1* and wild type mouse specimens. Expression was normalized with that of wild type. Data are presented as mean+/− SD for five mice from each genotype.

Diminished self-renewal of early mesenchymal progenitors in transgenic mice suggests that other mesenchymal lineages may be affected in a manner analogous to osteoprogenitors. Therefore, we studied adipogenesis *in vitro*. Consistent with the observed decrease in total stromal progenitors and osteoprogenitors, transgenic *mCol1α1-Pitx1* bone marrow (BM) stroma contained significantly fewer adipocyte progenitors than in wild type mice (Fig. 7A). Of note, morphological and gene expression analysis revealed that the adipocyte progenitors did not appear to mature normally (Fig. 7B). Given that defective mesenchymal stem or progenitor cell self-renewal was previously reported as age-dependent osteoporosis by Bonyadi *et al*., (2003) in *Sca-1/Ly6A-*null mice^10^, this prompted us to investigate a possible role of *Pitx1* in the transcriptional regulation of the *Sca-1/Ly6A* gene. Indeed, overexpression of Pitx1 (Fig. 8A) in transgenic mouse osteoblasts resulted in severe downregulation of endogenous *Sca-1/Ly6a* gene expression when compared to wild type mice osteoblasts (Fig. 8B).

### Osteoclastogenesis is affected by *Pitx1* overexpression in a non-cell autonomous manner

**Figure 9:**
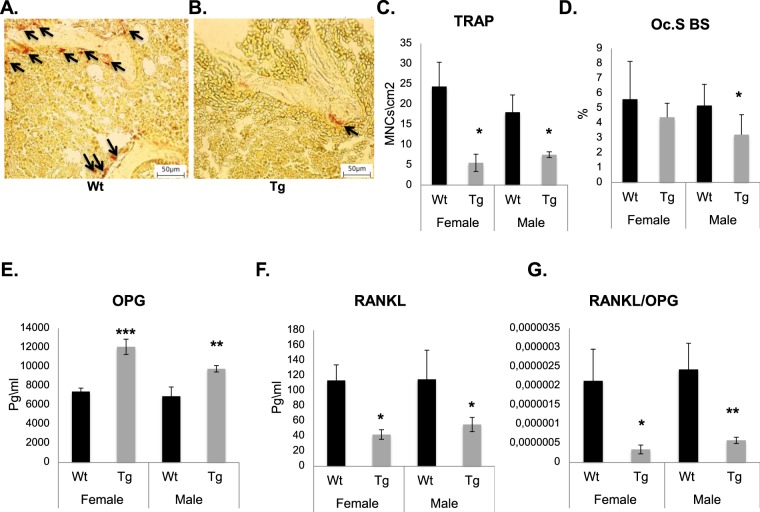
Impaired *in vivo* osteoclast number and function in *Col1α1-Pitx1* mice. (**A–C**) TRAP activity staining and quantitation in femur sections of both sexes from 12-week-old *Col1α1-Pitx1* and wild type mice. (**D**) Histomorphometric quantitation of the osteoclast surface (Oc.S BS) within trabecular bone areas. (**E–G**) Measurements of plasma levels of RANKL and OPG. Data are presented as mean ± SD for 5 mice from each sex in each genotype group. Asterisks indicate statistically significant difference (*p < 0.05, **p < 0.005).

Osteoblasts play a key role in the regulation of osteoclast function. Therefore, it is of interest to investigate whether *Pitx1* overexpression could interfere with the synthesis of paracrine osteoclast-regulating cytokines such as the receptor activator of nuclear factor kappa-B ligand (RANKL; encoded by *Tnfsf11* gene) and osteoprotegerin (OPG; encoded by *Tnfrsf11b* gene). Expression analysis showed a significant decrease in *Tnsfs11* (*Rankl*) expression in osteoblasts obtained from transgenic mice compared to those obtained from wild type mice (Fig. 8C). Conversely, expression of *Tnfrsf11b* (*Opg*) was increased in transgenic osteoblasts compared to wild type mouse osteoblasts (Fig. 8D). Histochemical evaluation revealed that the number of TRAP-positive osteoclasts in the trabecular bone decreased in transgenic mice by 77.3% in females (*P* = 0.013) and 58.3% in males (*P* = 0.037) compared to those in wild type mice (Fig. 9A–C). Histomorphometric analysis revealed a decrease in the percentage of the trabecular surface that is occupied by osteoclasts (Oc.S/BS) in transgenic mice (21.8% decrease in females and 37.3% decrease in males; *P* = 0.03) compared to wild type mice (Fig. 9D). Consistently, we observed a significant elevation in the levels of the plasma RANKL decoy receptor, osteoprotegerin (OPG) in transgenic mice (12070.1 ± 794 pg/ml, *P* < 0.001 in females and 9766.3 ± 794 pg/ml, *P* < 0.001 in males) compared to wild type mice (7392.5 ± 359 pg/ml, *P* < 0.001 in females and 6899.5 ± 973 pg/ml, *P* < 0.001 in males) (Fig. 9E). Moreover, there was a significant decrease in the levels of the osteoclastogenic cytokine RANKL in transgenic mice (42 ± 6.3 pg/ml, *P* < 0.01 in females and 55.2 ± 9.3 pg/ml, *P* < 0.01 in males) compared to wild type mice (113.4 ± 20.7 pg/ml, *P* < 0.01 in females and 115.1 ± 38.5 pg/ml, *P* < 0.001 in males) (Fig. 9F). This leads to a significant decrease in the RANKL/OPG ratio (Fig. 9G). Collectively, our data strongly suggest that Pitx1 overexpression in transgenic *mCol1α1-Pitx1* osteoblasts inhibits osteoclastogenesis in a non-cell autonomous manner.

### *Pitx1* overexpression inhibits the canonical Wnt/β-catenin signaling pathway

**Figure 10:**
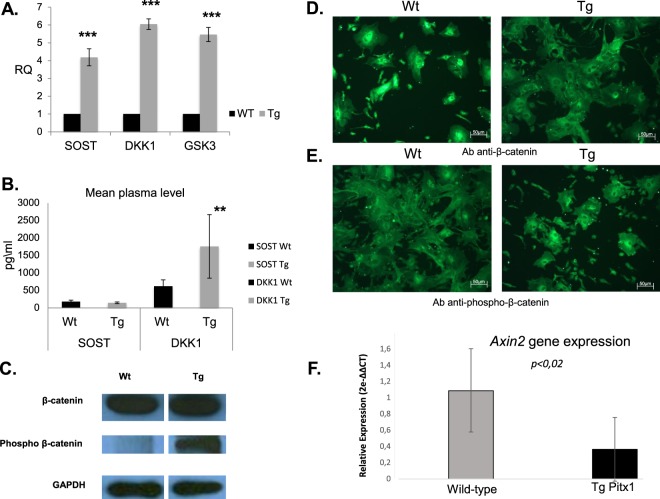
Inactivation of the Wnt canonical pathway in *Col1α1-Pitx1* mice. (**A**) Real time PCR analysis of Wnt canonical pathway markers sclerostin (SOST), Dickkopf-related protein 1 (DKK1), and glycogen synthase kinase-3β (GSK3β). Expression was normalized with the same proteins in wild type mice. (**B**) Mean plasma levels of SOST and DKK1 in *Col1α1-Pitx1* transgenic mice compared to that of wild type littermates. Asterisks indicate statistically significant difference (**p < 0.005; ***p < 0.0005). (**C**) Western blot of β-catenin *vs* phospho β-catenin obtained from protein extracts of cultured osteoblasts from *Col1α1-Pitx1* transgenic mice compared with their wild type littermates. (**D**) Immunolocalization of β-catenin by confocal microscopy with anti-β-catenin antibodies in wild type (Wt) and transgenic (Tg) mouse osteoblasts. (**E**) Detection of phospho-β-catenin by the same method with anti-phospho-β-catenin antibodies. Original uncropped blots corresponding to panel C are shown in Supplementary Fig. S4. (**F**) Real time qPCR expression analysis of *Axin-2*, a known Wnt target, was carried-out in transgenic m*Col1α1-Pitx1* and wild type mouse primary osteoblasts. Expression was normalized with that of wild type. Data are presented as mean+/− SD for five mice (females) from each genotype.

Osteoblast differentiation is predominantly regulated by the canonical Wnt/β-catenin signaling pathway, which, together with bone morphogenetic proteins (BMP), regulates osteogenesis. We were therefore interested in whether *Pitx1* overexpression could interfere with the Wnt/β-catenin signaling pathway. Expression analysis by quantitative real time PCR showed that the transcript levels of *Sost* and *Dkk1*, which are known inhibitors of Wnt signaling, increased by 4.1-fold (*P* = 0.005) and 6-fold (*P* = 0.0008), respectively, in transgenic mice osteoblasts compared to wild type mice cells (Fig. 10A). We further demonstrated a significant increase in plasma *Dkk1* levels in transgenic mice (1759 ± 908 pg/ml, *P* < 0.001) compared to wild type mice (621 ± 182 pg/ml, *P* < 0.001). No significant changes were observed in plasma *Sost* levels in transgenic mice (146 ± 24 pg/ml) compared to wild type mice (180 ± 43 pg/ml) (Fig. 10B). In addition, *Gsk3β* expression also increased 5.4-fold (*P* = 0.001) in transgenic osteoblasts compared to wild type osteoblasts (Fig. 10A). These results suggest that Pitx1 is a negative regulator of the canonical Wnt signaling pathway. Consistently, western blot analysis showed a significant increase in phospho-β-catenin in protein extracts of cultured transgenic osteoblasts compared to control cells (Fig. 10C). This was further illustrated at the cellular level by immunofluorescence and confocal microscopy showing the presence of β-catenin in the nuclei (strong signal) and cytosol of wild type mice osteoblasts, while β-catenin was only detected in the cytosol of transgenic osteoblasts (Fig. 10D). The same approach with anti-phospho-β-catenin antibodies revealed a stronger cytosolic accumulation of phospho-β-catenin in transgenic osteoblasts and confirmed our previous findings (Fig. 10E). Furthermore, we examined the expression of *Axin 2*, a well-known Wnt target, which was significantly downregulated in transgenic osteoblasts overexpressing *Pitx1* compared to wild type mouse osteoblasts (Fig. 10F). Collectively, our data confirm that *Pitx1* overexpression in transgenic mice may inactivate the canonical Wnt signaling pathway in osteoblasts by upregulating *Dkk1* at the mRNA and protein levels. Interestingly, transgenic *mCol1α1-Pitx1* mice treated with 0.01 M of lithium chloride (LiCl), a known GSK3-β inhibitor, were protected from bone loss compared to untreated transgenic mice (Supplementary Information and Figs S4 and S5). MicroCT (µCT) analysis showed significant improvements, mainly in the structural parameters of the trabecular bones of femurs harvested from transgenic mice treated with LiCl, when compared to untreated cells (Table 1).

**Table 1:** Lithium treatment of transgenic *mCol1α1-Pitx1* mice improves trabecular bone architecture parameters

	Tg *mCol1α1-Pitx1* (females)			Tg *mCol1α1-Pitx1* (males)		
	Treated with NaCl [0.01 M]	Treated with LiCl [0.01 M]	*P* values	Treated with NaCl [0.01 M]	Treated with LiCl [0.01 M]	*P* values
Number of mice	12	12		12	12	
**Trabecular Bone**						
Percent Bone Volume BV/TV (%)	9.0 ± 2.4	26.3 ± 8.7	<0.01	19.4 ± 6.1	37.1 ± 15.8	<0.01
Trabecular Pattern Factor Tb.Pf (1/mm)	27.8 ± 4.6	11.8 ± 4.6	<0.01	17.8 ± 6.3	2.5 ± 10.5	<0.01
Trabecular Thickness Tb.Th (mm)	0.07 ± 0.01	0.08 ± 0.01	0.01	0.06 ± 0.01	0.08 ± 0.02	0.02
Trabecular Separation Tb.Sp (mm)	0.3 ± 0.04	0.2 ± 0.05	<0.01	0.20 ± 0.01	0.16 ± 0.06	0.11
Bone Surface/Volume Ratio BS/BV (1/mm)	64.3 ± 6.2	49.7 ± 8.6	<0.01	58.4 ± 8.8	46.0 ± 14.2	0.01
Structure Model Index SMI	2.8 ± 0.2	1.5 ± 0.6	<0.01	2.0 ± 0.3	1.0 ± 0.7	<0.01
Trabecular Number Tb.N (1/mm)	1.3 ± 0.3	3.3 ± 0.9	<0.01	2.8 ± 0.5	4.3 ± 1.1	<0.01

## Discussion

Our data demonstrate that PITX1 regulates osteoblastogenesis and osteoclastogenesis in the bone. Using high-resolution microCT, we detected altered cortical and trabecular bone architecture in the long bones of transgenic *mCol1α1-Pitx1* mice to reveal a compromised microarchitecture of the bones. One striking feature of the transgenic phenotype is the altered mineralization due to the reduced quantity of bone matrix deposited by osteoblasts. There is a significant reduction in the mineral apposition rate and bone formation rate, which leads to decreased osteoid number and thickness. The altered mineralization and microarchitecture produce a mechanically weaker and immature bone, which could explain the reduced stiffness and strength of the transgenic bones. The significantly reduced expression of osteoblast differentiation markers further support these histomorphometric findings. Furthermore, increased *Sost* expression, a known osteocyte marker, in transgenic bone suggests an altered maturation rate of osteoblasts into osteocytes, which may explain the altered mineralization observed *in vivo* and *in vitro*^11^.

Interestingly, PITX1 also appears to contribute to the deregulation of the regulatory osteoblast-osteoclast coupling. In fact, overexpression of PITX1 in osteoblasts increased their capacity to inhibit osteoclastogenesis, by inducing the anti-osteoclastogenic factor, osteoprotegerin (OPG), as evidenced by the elevated plasma OPG levels and the reduced osteoclast number in transgenic bones compared to wild type bones. The parameters reflecting the structure and geometry of the trabecular bone clearly demonstrate the presence of more plate-like structures in transgenic bones (Fig. 2). This is likely the result of a concurrent decrease in osteoclast number and activity as well as impaired osteoblastogenesis. This phenotype is very similar to senile (type-II) osteoporosis, which differs from the observations of previous studies that link post-menopausal osteoporosis with rod-like trabecular structures and increased osteoclastogenesis^12^. Of note, acute lymphoblastic leukemia (ALL), the most common childhood malignancy, is often associated with decreased bone mineral density, which can lead to fractures, deformity and pain. Interestingly, Halton *et al*. previously reported a 16% prevalence of vertebral fractures in newly diagnosed childhood ALL cases^13^, while Nagel *et al*., reported a leukemic activation of PITX1 in 9% of ALL samples tested^14^. Although ALL is primarily a disease of the bone marrow and peripheral blood, any tissue or organ may be infiltrated by leukemic cells^15,16^. Consistently, histomorphometric studies of bone formation and resorption in newly diagnosed children with ALL showed a decrease in bone volume and trabecular thickness^17^. Moreover, a reduced number of both osteoblasts and osteoclasts with a subsequent decrease in osteoid formation and bone resorption were observed^17^. Despite the fact that the mechanism underlying osteoporosis development in childhood ALL around the time of diagnosis is still poorly understood, the discussed results ressemble those observed in our transgenic mice.

Despite several genome-wide association studies for osteoporosis and/or bone fracture, none of them reported a signal in/or around the PITX1 gene. By studying aging monozygotic twins discordant for osteoporosis, Mak *et al*., were the first to show an association between PITX1 overexpression and senile osteoporosis^1^. New advances in our understanding of the epigenetics of osteoporosis reveal additional mechanisms by which PITX1 expression could be deregulated in the context of osteoporosis. Indeed, many studies identified circulating microRNA signatures in men and women suffering from osteoporosis^18–20^. Among others, the expression of miR-19a-3p and miR-19b-3p is downregulated by about 2-fold in the serum of men suffering from idiopathic osteoporosis as well as in women with postmenopausal osteoporosis^18^. Both microRNAs are of interest given their capacity to bind within the 3′UTR of the *Pitx1* mRNA leading to its degradation and/or preventing its translation^21,22^. Furthermore, a recent study by Margolis *et al*., showed that circulating miR19b-3p expression is downregulated in aging male volunteers when compared to young ones^23^. It should be noted that miR-19a and miR-19b are both part of a cluster termed miR-17-92 whose haploinsufficiency in mice causes altered alkaline phosphatase (ALP) activity and reduced bone calcification^24^.

Another important observation of our study is that PITX1 may be a novel negative regulator of the canonical Wnt/β-catenin pathway through the upregulation of *Dkk1*, *Sost*, and *Gsk3β* expression, in transgenic osteoblasts, which results in increased phosphorylation of β-catenin. The upregulation and increased secretion of DKK1, a well-known biomarker of osteoporosis, is an important finding given it normally inhibits osteogenesis and promotes adipogenesis^25,26^. It is therefore, somewhat surprising that we did not detect greater bone marrow adiposity in transgenic mice. *Pitx1* overexpression reduced the number of MSCs and blocked osteogenesis and adipogenesis. The reduction in the number of MSCs could be explained by the fact that PITX1 is a known transcriptional repressor of telomerase reverse transcriptase (TERT)^21,27^, which plays a role in the survival, growth, and differentiation of mesenchymal stem cells (MSCs)^28^. However, no significant changes were detected in *mTert* expression in transgenic osteoblasts compared to wild type osteoblasts. Interestingly, overexpression of *Pitx1* led to a strong downregulation of *Sca-1/Ly6a* gene expression in transgenic osteoblasts, which is well known for its role in the regulation of mesenchymal progenitor self-renewal. However, it remains unclear, at this stage, exactly how *Pitx1* overexpression represses *Sca-1/Ly6* gene expression. Nevertheless, this role is strengthened by the work of Chen *et al*., (2009) which showed a clear dichotomy between subsets of pituitary stem/progenitor cells, where cells overexpressing Pitx1 were Sca-1-negative, while those that did not express Pitx1 were Sca-1 positive^29^.

Our experimental data and previous studies suggest that PITX1 exerts a dual action within osteoblast cell lineage; it represses the canonical Wnt/β-catenin signaling as well as the expression of *Sca-1/Ly6a*. Moreover, downregulation of *Sca-1/Ly6a* induced by *Pitx1* overexpression could explain the reduced MSC differentiation capacity as well as the maintenance of an undifferentiated state. Understanding the role of PITX1 in regulating multipotent MSC differentiation might open new avenues for the development of novel therapeutic strategies. Given that osteoporosis is a complex and heterogeneous disease, future studies will be required to confirm the contribution of PITX1 in human senile osteoporosis and more specifically, in some subsets of patients suffering from ALL or idiopathic osteoporosis.

## Materials and Methods

### Generation of transgenic *mCol1α1-Pitx1* mice

Transgenic C57BL/6 mice overexpressing *Pitx1* (*mCol1α1-Pitx1*) in pre-osteoblasts and osteoblasts were generated using a 2.3 kb fragment of the murine promoter for the Col1α1 protein^30^, to control the expression of the coding sequence of *Pitx1* (*Pitx1* murine cDNA), which was subcloned in a pCI plasmid. A synthetic intron was subcloned between the promoter and the coding sequence, and a polyadenylation cassette was subcloned downstream of the coding sequence in the same vector. The expression vector pCI-*mCol1α1-Pitx1* was injected into C57BL/6J ovocytes (IRIC’s transgenesis platform, Université de Montréal). The founders were screened for *Pitx1* overexpression by PCR using mouse tail genomic DNA. Transgene (TG) expression in tissues was assessed by RT-PCR using two sets of transgene-specific primers *Pitx1* (F: 5′-ACTGGGCTTGTCGAGACAGAG-3′; R: 5′-CAGATCAGCGTCGGACGATTC-3′). Four transgenic founders were identified. The transgene lines M22, F30, M42 and M51 were generated by mating founder animals with C57BL/6J mice. We selected the transgene line F30 for further characterization due to its high expression of *Pitx1* and its skeletal phenotype. Of note, all transgenic and wild type mice were maintained on a humid diet 5K52 (Lab Diet) since transgenic mice were prone to spontaneous fractures of the mandible and/or hind limbs while on the regular diet, usually deposited at the top of the cage. No significant adverse effect was observed using the humid diet. All the animal experiments were performed in accordance with the protocol (#519) approved by the Animal Ethics Committee of the CHU Sainte-Justine University Hospital Research Center. This study was conducted following the relevant regulations and guidelines.

### Radiologic imaging and dual beam X-ray absorptiometry

Ten transgenic mice (females = 5; males = 5) and ten wild type mice (females = 5; males = 5) of 12 weeks of age were analyzed with high resolution X-rays using Faxitron MX20 (Faxitron Corp, IL, USA). The bone mineral density (BMD) was measured with dual-energy X- ray absorptiometry (DXA; PIXIMus II densitometer; GE Healthcare, WI, USA) at 3, 5, and 7 months of age under anesthesia. Other time points were used when appropriate and are indicated.

### Microcomputed tomography analysis

The femoral bones of ten transgenic mice (females = 5; males = 5) and ten wild type mice (females = 5; males = 5) of 12 weeks of age were harvested and stored in 70% ethanol at 4 °C. The middle cortical and the trabecular bones of the distal metaphysis were scanned by microcomputed tomography (µCT) using a SkyScan-1072 desktop MicroCT scanner (Bruker microCT, Kontich, Belgium). The femoral bone samples were scanned by an X-Ray source power at 45 kV/222 µA and with 6.25 µm/pixel resolution. The rotation was set at 0.9 degrees per step for 180 degrees and the exposure time was set to 2.8 seconds for each step. The cross sections along the specimen axis were reconstructed using NRecon (v 1.6.1.3, SkyScan). A CT-Analyser (v 1.10.0.1, SkyScan) was employed to perform quantitative analyses and to create 3D models. 3D Creator (v 2.4, SkyScan) was used to manipulate the 3D models and to create 3D images.

### Image analyses with ImageJ software

The mean color values of safranin O staining was determined from averaging 7 measurements each of stained histological sections from transgenic and wild type mice. For each measurement, a region of interest (a rectangle) was drawn on the image using ImageJ and the mean pixel intensity (black pixel = 0 and white pixel = 255) of the region of interest (ROI) was measured. These ROIs were drawn around the growth plate matrix (no cell). To obtain the intensity of the staining, the measurements were subtracted from 255. This analysis was also performed for the background and its value was subtracted from the staining intensity value. The mean and standard deviation were automatically calculated from the values of the pixels within the ROI. The same method was used to quantify changes in mineralized bone nodules stained with alizarin red.

### Bone material and biomechanical properties

Freshly dissected femurs of ten transgenic mice (females = 5; males = 5) and ten wild type mice (females = 5; males = 5) of 12 weeks of age were tested for their biomechanical strength using a three-point bending test performed on a Mach-1^TM^ micro-mechanical testing system (Biosyntech Inc., Quebec, Canada). Extrinsic parameters, such as ultimate force (N), stiffness (N/mm), and work to ultimate point (N*mm) were obtained from a force displacement curve. The displacement resolution was 0.1 µm, the travel range was 25 mm, the ramp velocity was 50 µm/s, the load cell resolution was 0.5 g for ± 10 kg, and the fixed relaxation time was 60 s.

### Preparation of primary osteoblasts and mesenchymal stem cells

Osteoblasts and mesenchymal stem cells (MSCs) were isolated from five transgenic mice and five wild type mice of 12 weeks of age for each sex. The femurs were dissected and thoroughly cleared of muscle and connective tissue, after which the outer surfaces of the bone were scraped to remove the periosteal surface. The bone marrow was flushed with 5 ml media. Large fragments were removed by filtration using a 70-µm nylon mesh cell strainer. The cells were pelleted by centrifugation and resuspended in 2 ml media. The cells were plated at a density of 2 × 10^5^ cells/cm^2^ in 6-well microplates with Alpha Modification Eagle’s Medium 1 × (alpha-MEM) (Wisent Inc., St-Bruno, QC, Canada) containing 20% fetal bovine growth serum (FBS) (GE Healthcare Life Sciences, UT, USA) and 1% antibiotic-antimycotic solution (Thermo Fisher Scientific Inc., NY, USA). The bones were cut into small pieces of 1–3 mm^3^ using a sterile stainless steel surgical blade. The pieces of bone were washed in serum-free media three times and then serially digested twice by incubating in 1 mg/ml type-I collagenase for 20 min followed by 2 hours of further incubation (Sigma-Aldrich Canada Co.) at 37 °C. The digested bones were washed three times in serum-free medium, with vigorous hand shaking and then cultured in alpha-MEM with 20% FBS and 1% antibiotic-antimycotic solution. Osteoblast differentiation of primary osteoblasts or MSCs was induced by culturing the cells in alpha-MEM media supplemented with 10% FBS, 100 nM dexamethasone, 20 mM glycerophosphate, 50 μM ascorbate-2-phosphate amd 50 nM vitamin D3.

### Alkaline phosphatase and alizarin red staining of osteoblast cultures

For alkaline phosphatase (ALP) staining, the cells were fixed with 4% paraformaldehyde (PFA) for 2 minutes, stained with ALP detection solution (Sigma-Aldrich Canada Co.) for 4 minutes at 37 °C and fixed again with 4% PFA for an additional 10 minutes. The mineralized osteoblast cultures were fixed in 70% ethanol for 1 hour at 4 °C. The cells were then stained with 0.2% Alizarin red in 2% ethanol for 15 minutes. The cells were washed five times with water and dried at 37 °C. Mesenchymal stem cells (MSCs) were fixed in 4% PFA, stained for Alizarin red, photographed and counterstained with hematoxylin for colony quantitation.

### Adipocyte differentiation assay

The MSCs were plated at a density of 2 × 10^5^ cells/cm^2^ in 6-well microplates in alpha-MEM (10% FBS, 1% antibiotic-antimycotic solution). To induce the production of adipocytes, the medium was supplemented with 1 µM dexamethasone, 5 µg/ml insulin, 50 µM indomethacine, and 0.5 µM isobutyl methylxanthine. The media was changed every 4 days over a 2-week period, and the adipocytes were then visualized using oil red ‘O’ staining.

### Isolation of RNA and protein from bone tissues, real time PCR, and Western blotting

The long bone tissues of five transgenic mice and five wild type mice of 12 weeks of age of each sex were isolated from all the soft tissues, and the bone marrow was removed by flushing with PBS. The bone samples were then pulverized using liquid nitrogen and a frozen mortar and pestle. Total RNA was extracted using TRIZOL reagent (Thermo Fisher Scientific Inc.) and cDNA was prepared using the ThermoScript™ RT-PCR kit (Thermo Fisher Scientific Inc.). Real time PCR was performed in the Mx3000P PCR (Stratagene Corp, CA, USA) using the QuantiTect® SYBR® Green PCR kit (Qiagen Inc., ON, Canada) to detect the mRNA expression of *Pitx1*, *Runx2*, *Osx/Sp7*, *Alp1*, *Ocn*, *Spp1*, *Ppargamma-1*, *Cebp*, *Adipoq*, *Ap2*, *DKK1*, *SOST*, and *GSK3-β*. The specific DNA primers used are shown in Supplementary Table S1. All real time PCR experiments were performed in triplicate, and β-actin was used as a housekeeping gene control. The ΔΔCT method was used for qPCR data analysis. Total protein lysates were obtained from cultured osteoblasts of transgenic *mCol1α1-Pitx1* and wild type mice. Western blotting was performed to detect the expression of β-catenin and phospho-β-catenin in the bone lysate by probing with either rabbit anti-β-catenin antibody (ab6302) (1:4000 dilution) or rabbit anti-phospho-β-catenin (ab27798) (1:500 dilution) or mouse anti-GAPDH (sc-20357) (1:5000), followed by horseradish peroxidase (HRP)-conjugated anti-rabbit (eBioscience,18-8816-33) or anti-mouse (eBioscience,18-8817-33) secondary antibody (1:5000), respectively.

### Knockdown and overexpression of *Pitx1*

Knockdown of *Pitx1* expression was performed in primary *Col1α1-Pitx1* osteoblasts transfected with a scrambled sequence or *Pitx1* siRNA oligonucleotide (5′-AAACGACGAGUGCUGUUUGGACUUG-3′) (Thermo Fisher Scientific Inc.), using Lipofectamine RNAiMAX (Thermo Fisher Scientific Inc.). Overexpression of *Pitx1* was performed by infecting primary wild type mouse osteoblasts with lenti-X^TM^ Lentivirus Expression Systems (Clontech Laboratories, Inc., CA, USA).

### Quantification of plasma biomarkers

The levels of *RANKL*, *OPG*, *DKK1*, and *SOST* in plasma were measured using ELISA kits for RANKL, OPG (Abcam®,Toronto, Canada), DKK1, and SOST (R&D Systems, Inc., Minneapolis, USA).

### Expression analysis by quantitative real time PCR

Expression analysis by qPCR for *Pitx1*, *Sca-1 (Ly6a)*, *Tnfsf11*, *Tnfrsf11b*, *and Axin2* was performed using TaqMan gene expression probes for mice with *Gapdh* as an endogenous control (*Pitx1* #Mm00440824_m1; *Ly6a* #Mm00726565_s1*; Tnfsf11* #Mm00441906_m1*; Tnfrsf11b* #Mm00435454_m1*; Axin2* #Mm00443610_m1; *Gapdh #*Mm99999915_g1). The TaqMan Fast Advanced mastermix was used by following the manufacturer’s instructions (Thermo Fisher Scientific). Analysis was performed using the 7500-Fast Real Time instrument (Thermo Fisher Scientific). The ΔΔCT method was used for qPCR data analysis.

### Statistical analysis

The data were evaluated statistically using Student’s *t* test in SPSS Windows, version 10.0. Results are shown as mean ± standard deviation (SD). The p values were considered significant at *p < 0.05, **p < 0.01, and ***p < 0.001.

## Supplementary information

Bone-Specific Overexpression of PITX1 Induces Senile Osteoporosis in Mice Through Deficient Self-Renewal of Mesenchymal Progenitors and Wnt Pathway Inhibition
