# Supplementary material for: Bone-Specific Overexpression of PITX1 Induces Senile Osteoporosis in Mice Through Deficient Self-Renewal of Mesenchymal Progenitors and Wnt Pathway Inhibition
Source: Sci Rep. 2019 Mar 5;9:3544. doi: 10.1038/s41598-019-40274-6 (PMC6401072; doi:10.1038/s41598-019-40274-6)
Supplement: Supplementary file 1 — Bone-Specific Overexpression of PITX1 Induces Senile Osteoporosis in Mice Through Deficient Self-Renewal of Mesenchymal Progenitors and Wnt Pathway Inhibition [file 41598_2019_40274_MOESM1_ESM.pdf]

Bone-Specific Overexpression of PITX1 Induces Senile Osteoporosis in Mice Through Deficient Self-Renewal of Mesenchymal Progenitors and Wnt Pathway Inhibition

Nancy Karam[1,2], Jean François Lavoie[1,2], Benoit St-Jacques[1], Saadallah Bouhanik[1], Anita Franco[1], Nihad Ladoul[1,3] and Alain Moreau[1,2,3,*]

[1]Viscogliosi Laboratory in Molecular Genetics of Musculoskeletal Diseases, Sainte-Justine University Hospital Research Center, Montréal, Québec, H3T 1C5, Canada; [2]Department of Biochemistry and Molecular Medicine, Faculty of Medicine, Université de Montréal, Montréal, Québec, H3T 1J4, Canada; [3]Department of Stomatology, Faculty of Dentistry, Université de Montréal, Montréal, Québec, H3T 1J4, Canada.

*Correspondence and requests for materials should be addressed to A.M. (email: alain.moreau@recherche-ste-justine.qc.ca).

Materials and Methods

Calcein labeling and dynamic bone histomorphometry

Ten transgenic mice (females = 5; males = 5) and ten wild type mice (females = 5; males = 5) of 12 weeks of age were injected with 2 intraperitoneal calcein injections (10 mg/kg body weight) (Sigma- Aldrich Canada Co., ON, Canada) at 10 and 3 days prior to sacrifice. The right femurs and tibias were fixed with 70% ethanol overnight and dehydrated in a graded ethanol series. The bones were embedded in methyl methacrylate (MMA) for analyzing bone formation parameters. Mid-frontal sections of the distal tibia and femur with 6 μm thickness were used for toluidine blue staining or Goldner's trichrome staining. Sections of 10 μm thickness were used for fluorescence observation. The left femurs and tibias were fixed in 4% paraformaldehyde (PFA) at 4°C overnight

and decalcified before embedding in paraffin. The 6 µm- thick sections were stained for osteoclasts using tartrate-resistant acid phosphatase (TRAP) (Sigma-Aldrich Canada Co.). OsteoMeasure (Osteometrics, Inc., GA, USA) bone analysis software was used for histomorphometric analyses to measure the mineralized surface to bone surface ratio (MS/BS), bone formation rate to bone surface ratio (BFR/BS), and the mineral appositional rate (MAR).

Expression analysis by quantitative real-time PCR. Quantitative real-time PCR was performed in an Mx3000P PCR (Stratagene Corp, CA, USA) using the QuantiTect® SYBR® Green PCR kit (Qiagen Inc., ON, Canada) to detect the mRNA expression of *Pitx1, Runx2, Osx/Sp7, Alp1, Ocn, Spp1, Ppargamma-1, Cebp, Adipoq, Ap2, DKK1, SOST*, and *GSK3-β*. The specific DNA primers used are shown in the Supplementary Table S1. All real-time PCR experiments were performed in triplicate and β-actin was used as a housekeeping gene control. The $\Delta\Delta C_T$ method was used for qPCR data analysis.

Lithium treatment. A group of 12 transgenic males and 12 transgenic females received only 0.01 M NaCl (control group) diluted in water for 2 months, and the second group of 12 transgenic males, and 12 transgenic females received 0.01 M LiCl (Sigma catalog no. L9650) diluted in water for 2 months.

Supplementary Figure and Table Legends

Supplementary Figure S1. Comparison of changes in bone mineral density (BMD) and bone mineral content (BMC) between transgenic *mCol1α1-Pitx1* mice and wild-type mice. A-B. Total bone mineral density (BMD) (g/cm2) and **C-D**, bone mineral content (BMC) (grams) were determined by DEXA scan for female and male transgenic *m Col1α1-Pitx1* mice and their corresponding wild type littermates during growth over a period of 28 weeks. Data are presented as mean +/- SD for 5 mice from each sex in each genotype group. Asterisks indicate statistically significant difference (*$p < 0.05$).

Supplementary Figure S2. Trabecular bone volume and trabecular number. The trabecular bone volume (Tb.BV/TV) (A) and trabecular number (Tb.N) (B) were measured in 12-week-old transgenic *mCol1α1-Pitx1* and wild type mice. The difference in Tb.BV/TV was not significant (irrespective of sex) between transgenic and wild type mice (females $P = 0.293$ and males $P = 0.090$, respectively). The difference in Tb.N was also not significant (irrespective of sex) between transgenic and wild type mice (females $P = 0.080$ and males $P = 0.340$, respectively).

Supplementary Figure S3. Impaired *in vivo* osteoblast function derived from 12-week-old transgenic *mCol1α1-Pitx1* mice. A-B. Dynamic histomorphometric analysis of femurs from 12-week-old transgenic *mCol1α1-Pitx1* and wild type mice. **C-E.** Dynamic histomorphometric quantitation of the bone formation rate (BFR/BS), mineral apposition rate (MAR) and the mineralizing surface (MS/BS). Data are presented as mean +/- SD for five mice from each sex in each genotype. Asterisks indicate statistically significant difference (*p < 0.05; **p < 0 .005).

Supplementary Figure S4. Treatment with LiCl improves trabecular microarchitecture of female transgenic *mCol1α1-Pitx1* mice. A-C. Representative cross-sectional microCT scans of femurs from untreated 12-week-old female transgenic *mCol1α1-Pitx1*. **D-F.** Representative cross-sectional microCT scans of femurs from LiCl-treated 12-week-old female transgenic *mCol1α1-Pitx1*.

Supplementary Figure S5. Treatment with LiCl improves trabecular microarchitecture of male transgenic *mCol1α1-Pitx1* mice. A-C. Representative cross-sectional microCT scans of femurs from untreated 12-week-old male transgenic *mCol1α1-Pitx1*. **D-F.** Representative cross-sectional microCT scans of femurs from LiCl-treated 12-week-old male transgenic *mCol1α1-Pitx1*.

Supplementary Figure S6. Western blot analysis of phospho β-catenin vs β-catenin. Original uncropped western blots of phospho β-catenin (**A**), β-catenin (**B**), and GAPDH (**C**), obtained from protein extracts of cultured osteoblasts of *Col1α1-Pitx1* transgenic

mice and compared with those of their wild-type littermates. 25 µg or 40 µg total protein was loaded into each well. In panel C, the band seen in the second well with 40 µg total protein is an artefact caused by an error of manipulation of the sample that led to the loading of an extra well.

Supplementary Table S1. Oligonucleotides used for quantitative real-time PCR

Gene	Nucleotide Sequence
Pitx1	F: 5′- GCCTCAACCCGTGAACTGAA - 3′<br>R: 5′- GCGTAAACTCCAGCAGTGAT- 3′
Runx2	F: 5′- GCTATTAAAGTGACAGTGGACGG - 3′<br>R: 5′- GGCGATCAGAGAACAAACTAGG - 3′
Alp1	F: 5′ - ACTGATGTGGAATACGAACTGG - 3′<br>R: 5′ - AGTTCAGTGCGGTTCCAG - 3′
Sp7	F: 5′- CCCAGCTCGAGGATGGCGTC - 3′<br>R: 5′- AGGGAGCTGGGTAGGCGTCC - 3′
Ocn	F: 5′- AGATGCCAAGCCCAGCGGC - 3′<br>R: 5′ - CCTGCACGTCTAGCCCTCTG - 3′
Spp1	F: 5′ - GTGATTTGCTTTTGCCTGTTTG - 3′<br>R: 5′ - GAGATTCTGCTTCTGAGATGGG - 3′
Pparγ	F: 5′ - ATAGGTGTGATCTTAACTGCCG - 3′<br>R: 5′ - CCAACAGCTTCTCCTTCTCG - 3′
Cebp	F: 5′ - CATGCCGGGAGAACTCTAAC - 3′<br>R: 5′ - CTGGAGGTGACTGCTCATC - 3′
Adipoq	F: 5′ - TGTCTGTACGATTGTCAGTGG - 3′<br>R: 5′ - GCAGGATTAAGAGGAACAGGAG - 3′
Ap5	F: 5′ – GACAGGAAGGTGAAGAGCATC - 3′<br>R: 5′ – GTCACGCCTTTCATAACACATTC - 3′
SOST	F: 5′ - ACAACCAGACCATGAACCG - 3′<br>R: 5′ - CAGGAAGCGGGTGTAGTG - 3′
DKK1	F: 5′ - CACCATCAAGCCAGCAATTC - 3′<br>R: 5′ - CTGAAGATTCCTACATCCTTGGG - 3′
Gsk3-β	F: 5′ - CACCTGCACTCTTCAACTTTAC - 3′<br>R: 5′ - CACGGTCTCCAGCATTAGTATC - 3′
β actin	F: 5′ - ACCTTCTACAATGAGCTGCG - 3′<br>R: 5′ - CTGGATGGCTACGTACATGG - 3′

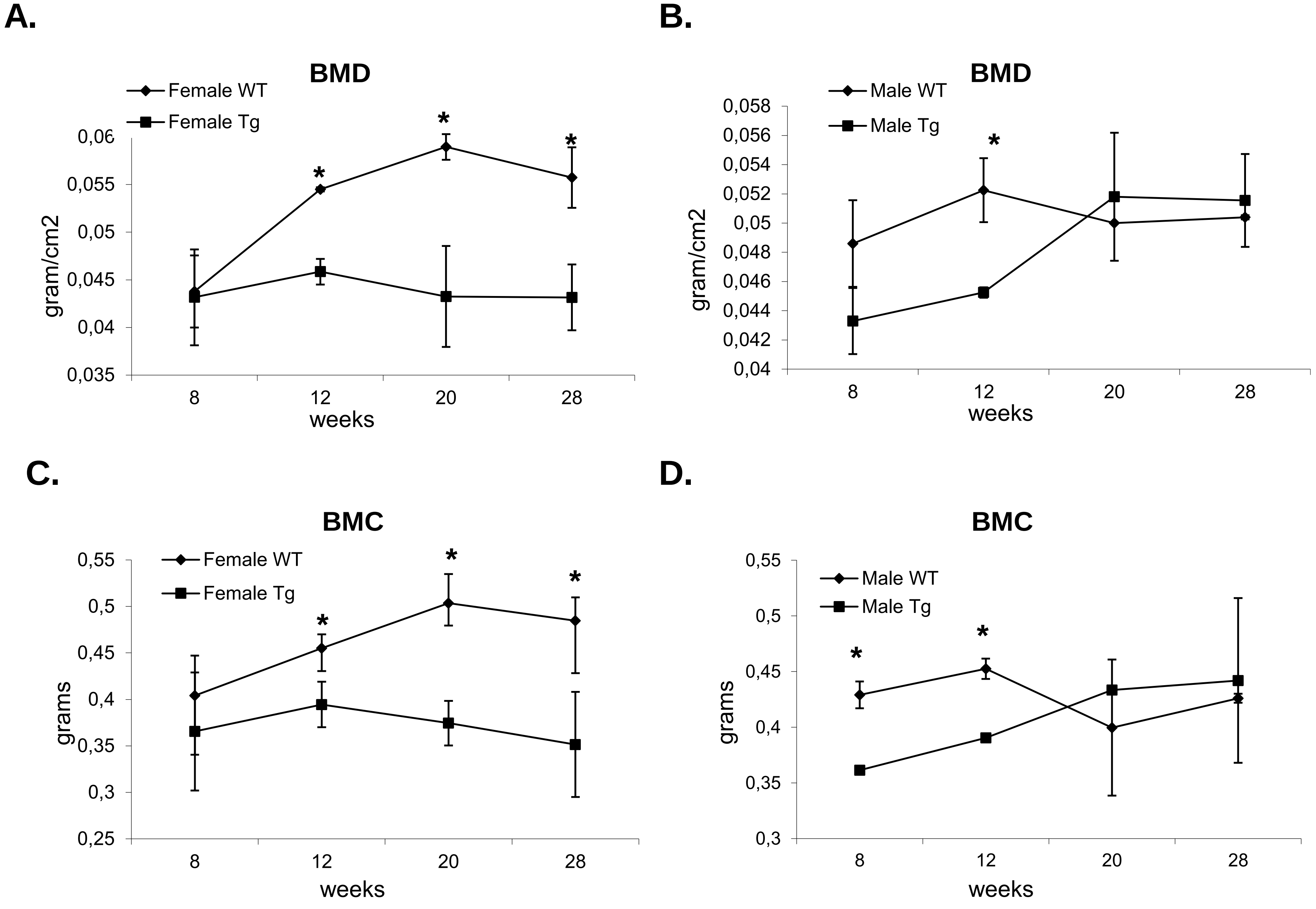
A.
BMD
Female WT
Female Tg
gram/cm2
0,06
0,055
0,05
0,045
0,04
0,035
8
12
20
28
weeks
B.
BMD
Male WT
Male Tg
gram/cm2
0,058
0,056
0,054
0,052
0,05
0,048
0,046
0,044
0,042
0,04
8
12
20
28
weeks
C.
BMC
Female WT
Female Tg
grams
0,55
0,5
0,45
0,4
0,35
0,3
0,25
8
12
20
28
weeks
D.
BMC
Male WT
Male Tg
grams
0,55
0,5
0,45
0,4
0,35
0,3
8
12
20
28
weeks

Figure S1

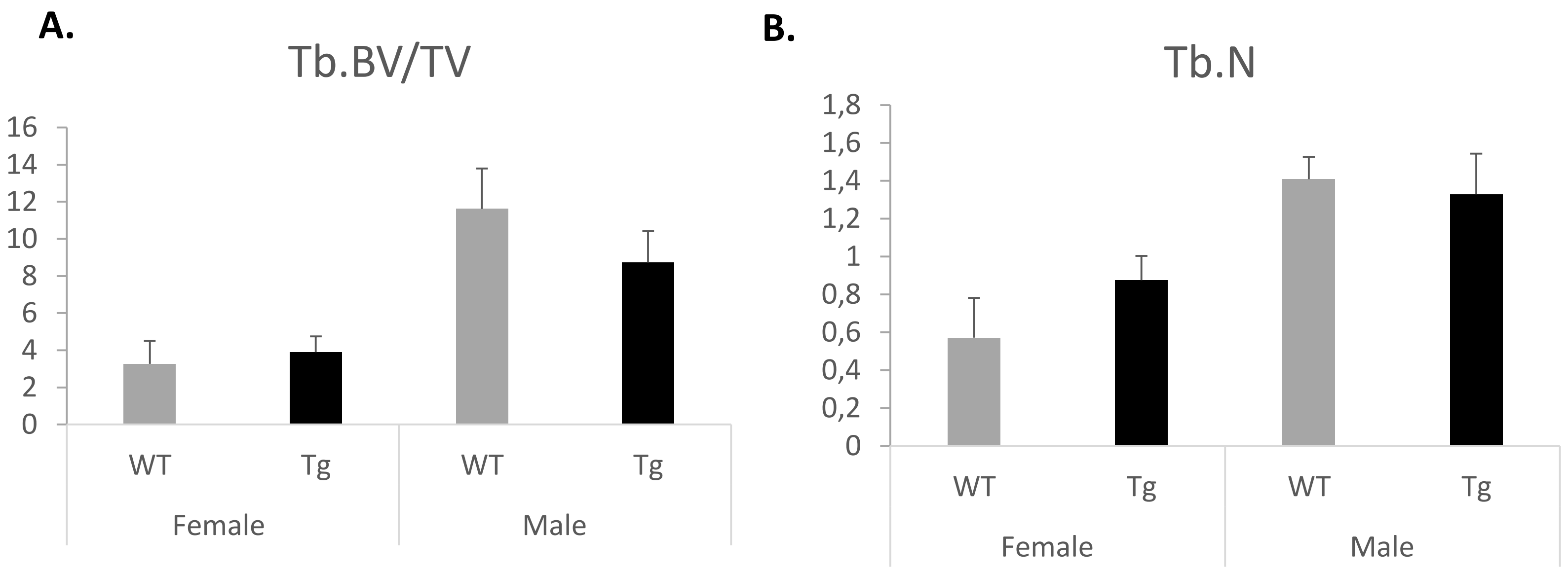
A.
Tb.BV/TV
16
14
12
10
8
6
4
2
0
WT
Tg
WT
Tg
Female
Male
B.
Tb.N
1,8
1,6
1,4
1,2
1
0,8
0,6
0,4
0,2
0
WT
Tg
WT
Tg
Female
Male

Figure S2

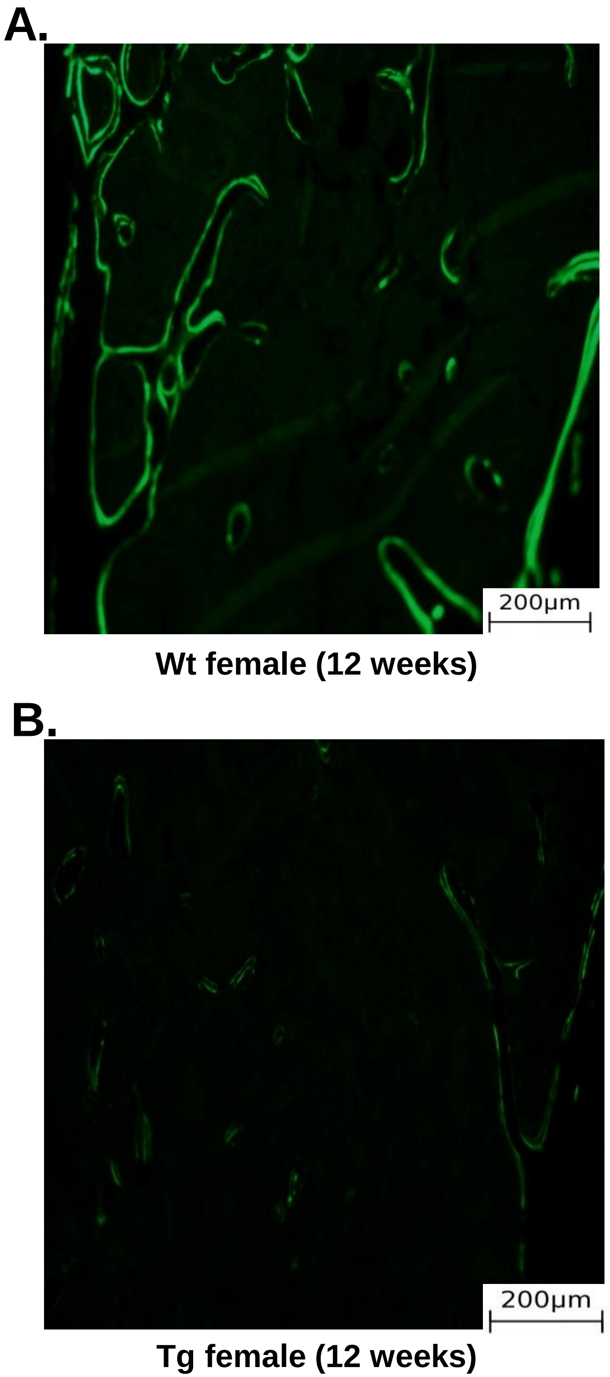
A.
200µm
Wt female (12 weeks)
B.
200µm
Tg female (12 weeks)

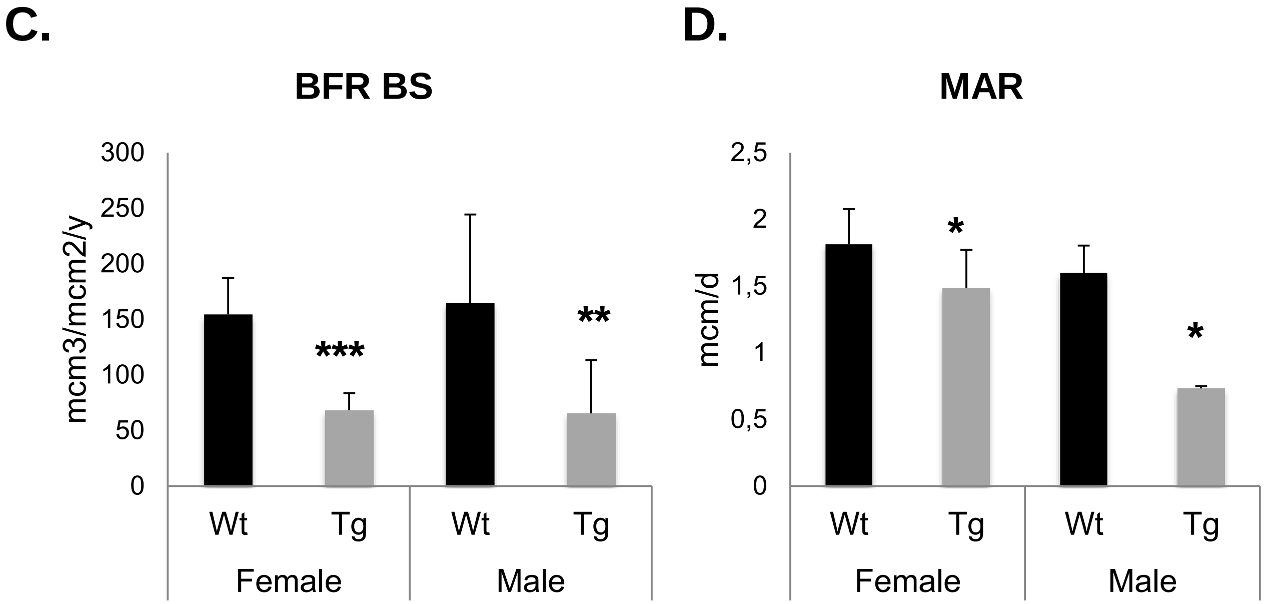
C.
BFR BS
mcm3/mcm2/y
300
250
200
150
100
50
0
***
**
Wt
Tg
Wt
Tg
Female
Male
D.
MAR
mcm/d
2,5
2
1,5
1
0,5
0
*
*
Wt
Tg
Wt
Tg
Female
Male

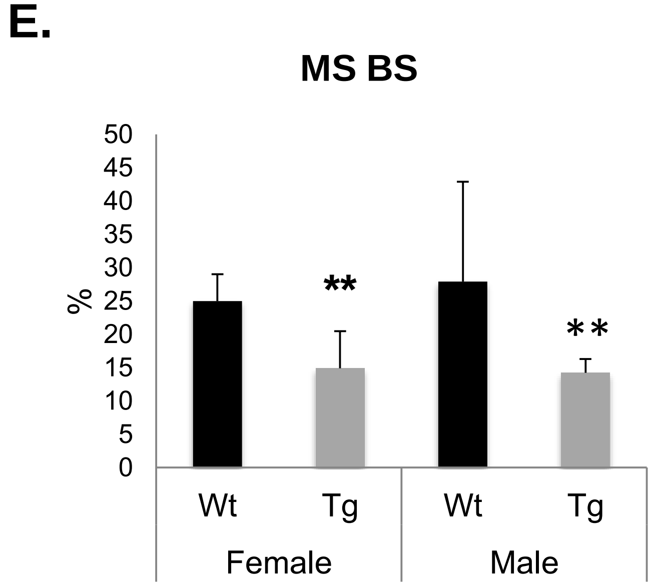
E.
MS BS
%
50
45
40
35
30
25
20
15
10
5
0
**
**
Wt
Tg
Wt
Tg
Female
Male

Figure S3

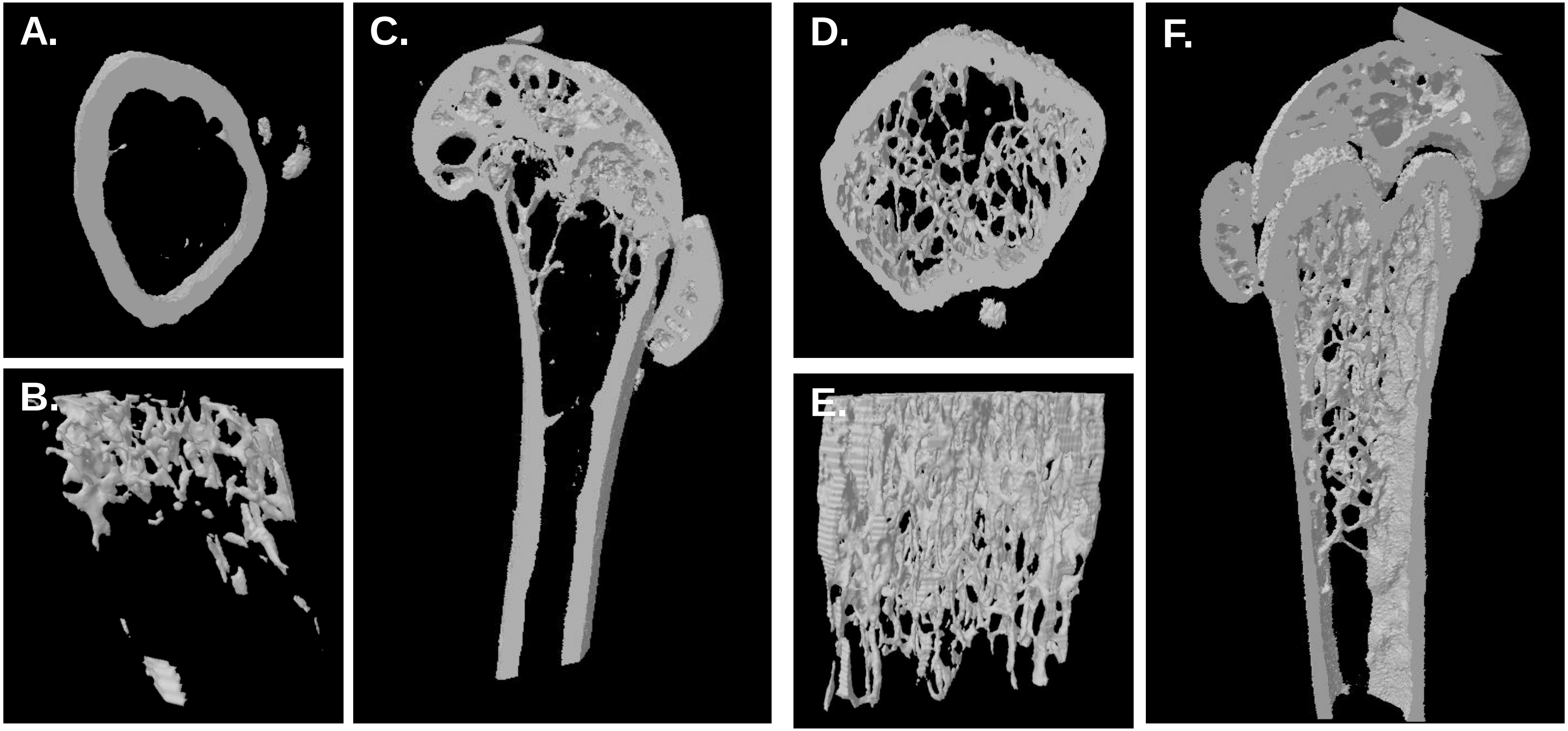

F-Tg *mCol1a1-Pitx1*-2268-Ct
12 weeks

F-Tg *mCol1a1-Pitx1*-2295-LiCl
12 weeks

Figure S4

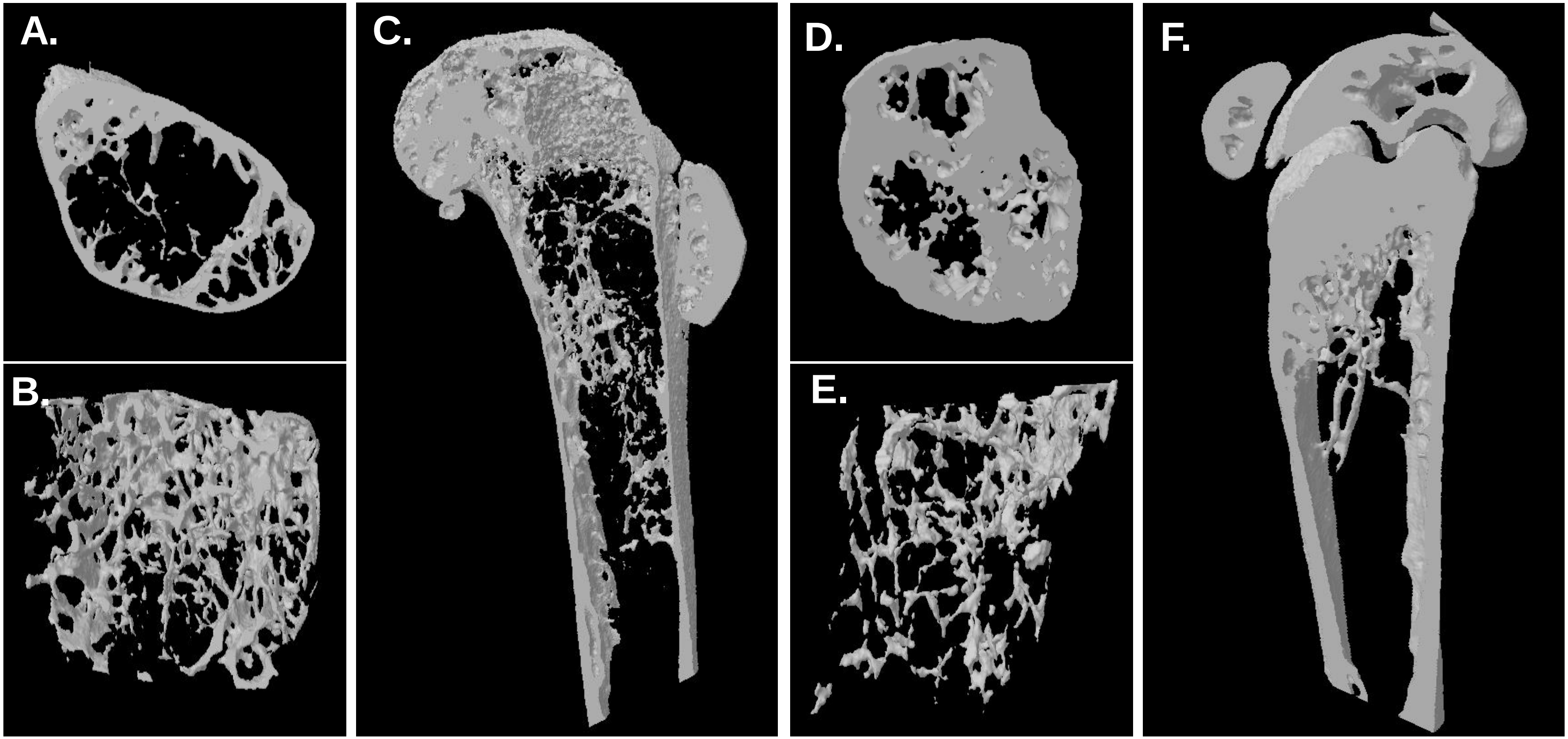

M-Tg *mCol1a1-Pitx1*-2266-Ct
12 weeks

M-Tg *mCol1a1-Pitx1*-2289-LiCl
12 weeks

Figure S5

Figure S6

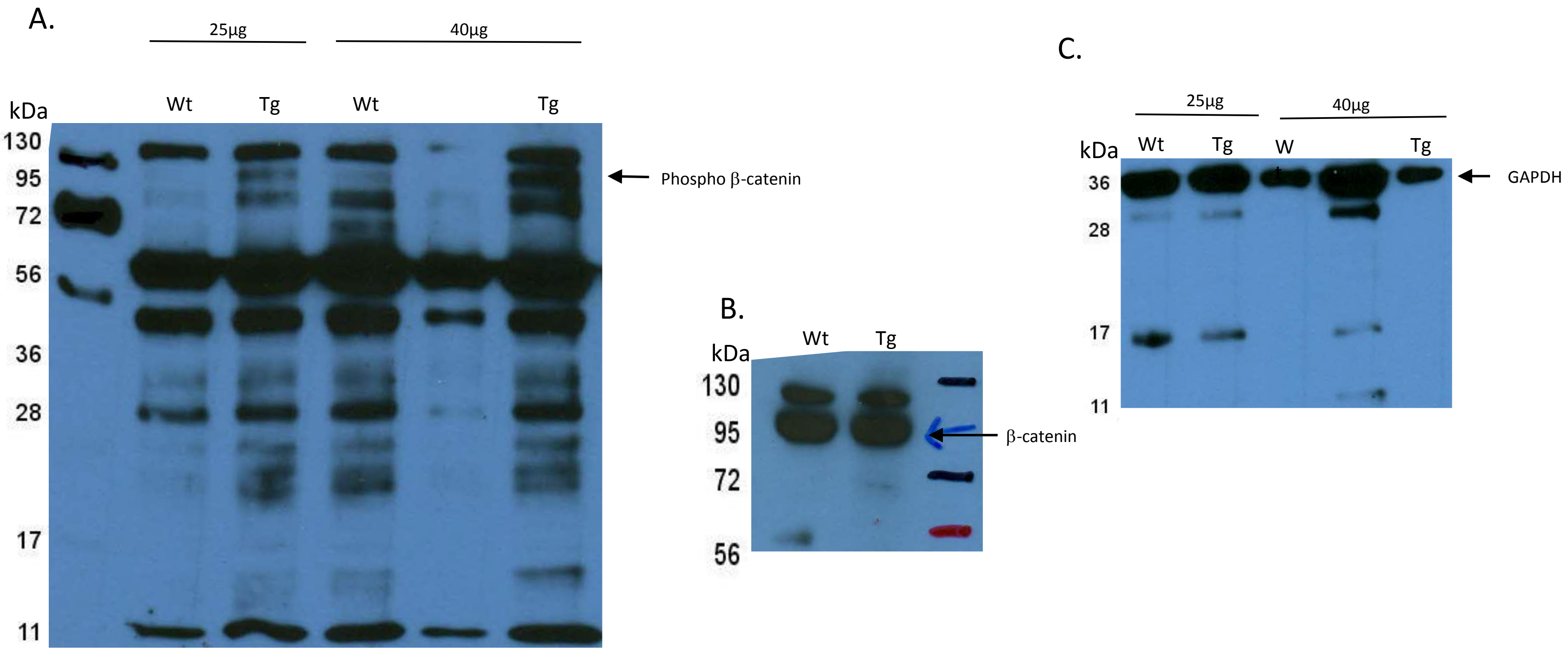
A.
25µg
40µg
Wt
Tg
Wt
Tg
kDa
130
95
72
56
36
28
17
11
Phospho β-catenin
B.
Wt
Tg
kDa
130
95
72
56
β-catenin
C.
25µg
40µg
kDa
Wt
Tg
W
Tg
36
28
17
11
GAPDH
